# Evaluation of Laser Therapy During Orthodontic Treatment in Adult Patients by Determining N-Telopeptide Levels in Gingival Crevicular Fluid

**DOI:** 10.3390/medicina61122170

**Published:** 2025-12-05

**Authors:** Luminița Lazăr, Dora Maria Popescu, Timea Dakó, Mihaela Moisei, Dorin Nicolae Gheorghe, Anamaria Bud, Mădălina Oprica, Mariana Păcurar, Ana-Petra Lazăr

**Affiliations:** 1 Department of Periodontology, George Emil Palade University of Medicine, Pharmacy, Science, and Technology of Targu Mures, 38 Ghe. Marinescu Street, 540139 Târgu Mures, Romania; luminita.lazar@umfst.ro; 2 Department of Periodontology, Faculty of Dental Medicine, University of Medicine and Pharmacy of Craiova, 2 Petru Rares Street, 200349 Craiova, Romania; popescudoramaria@yahoo.com (D.M.P.);; 3 Department of Odontology and Oral Pathology, George Emil Palade University of Medicine, Pharmacy, Science, and Technology of Targu Mures, 38 Ghe. Marinescu Street, 540139 Târgu Mures, Romania; 4 Department of Dentistry, Faculty of Medicine and Pharmacy, “Lower Danube” University of Galați, 35 Al.I. Cuza Street, 800010 Galați, Romania; 5 Department of Pedodontics, George Emil Palade University of Medicine, Pharmacy, Science, and Technology of Targu Mures, 38 Ghe. Marinescu Street, 540139 Târgu Mures, Romania; anamaria.bud@umfst.ro; 6 Immunology Laboratory, Center for Advanced Medical and Pharmaceutical Research, George Emil Palade University of Medicine, Pharmacy, Science, and Technology of Targu Mureș, 38 Ghe. Marinescu Street, 540142 Târgu Mures, Romania; madalina.nedelcu@umfst.ro; 7 Department of Orthodontics, George Emil Palade University of Medicine, Pharmacy, Science, and Technology of Targu Mures, 38 Ghe. Marinescu Street, 540139 Târgu Mures, Romania; mariana.pacurar@umfst.ro; 8 Department of Oral Rehabilitation and Occlusology, George Emil Palade University of Medicine, Pharmacy, Science, and Technology of Targu Mures, 38 Ghe. Marinescu Street, 540139 Târgu Mures, Romania; ana.lazar@umfst.ro

**Keywords:** N-telopeptide, bone remodeling, laser therapy, orthodontic treatment

## Abstract

*Background and Objectives*: Orthodontic tooth movement triggers micro-trauma in the periodontal ligament, leading to a balanced process of bone resorption and apposition mediated by local inflammatory responses. Monitoring N-telopeptide levels in gingival crevicular fluid (GCF) and applying low-intensity laser biostimulation can help optimize mechanical loading, reduce adverse effects, and enhance tissue remodeling during treatment. *Materials and Methods*: This study had a split-mouth observational design. From 30 patients with ages between 20 and 50, with standardized fixed orthodontic treatment, GCF samples were collected from both control and laser-treated hemiarches before and 14 days after appliance activation. Low-intensity laser therapy (LLLT) was applied to selected sites to assess its effect on N-telopeptide levels, a marker of bone resorption, with samples analyzed via ELISA and results compared statistically to evaluate the impact of laser biostimulation during orthodontic treatment. Statistical analysis was performed using paired *t*-tests or Wilcoxon tests for two-group comparisons. *Results*: N-telopeptide levels in gingival crevicular fluid increased significantly from baseline (T0) to 14 days (T1) in both the laser-treated (HL) and control (sham) hemiarches (HC), with higher values observed in the lasered side. Statistical analysis confirmed significant differences between HL and HC at T1 (*p* < 0.0001), as well as between each T1 group and baseline, indicating that low-intensity laser therapy enhanced bone resorption activity during orthodontic tooth movement. *Conclusions*: N-telopeptide exhibited higher values in the hemiarches where laser therapy was applied than in the control ones. This provides a rationale for using laser biostimulation as an adjuvant during orthodontic treatment to modulate tissue restructuring.

## 1. Introduction

In recent years, the proportion of adult patients seeking orthodontic treatment has steadily increased. Orthodontic treatment in adults differs significantly from that in children and adolescents. In growing individuals, many malocclusion traits can be addressed by influencing physiological growth through the use of orthopedic appliances. Adult patients, lacking growth potential, require alternative treatment protocols that primarily focus on dentoalveolar compensation. As a result, orthodontic treatment in adults may vary in terms of expectations, duration, and achievable outcomes. Furthermore, orthodontic tooth movement is fundamentally a biological process initiated by mechanical forces that are translated into biochemical signals, relying heavily on the physiology of both mineralized and non-mineralized tissues [1].

Orthodontic tooth movement induces micro-trauma to the periodontal ligament, associated with a cascade of local inflammatory phenomena that allows tooth movement and can be described as a continuous and balanced process of bone apposition and resorption. Under the action of orthodontic forces, bone resorption in the pressure zone and bone formation in the tension zone of the periodontal ligament occur simultaneously, thus contributing to the controlled movement of teeth during orthodontic treatment [2,3,4,5].

Many studies have focused on identifying biomarkers present in gingival crevicular fluid (GCF), with the aim of monitoring these processes dynamically and non-invasively [6,7,8,9]. Numerous protein biomarkers or protein derivatives are present in gingival crevicular fluid, which are released during bone remodeling by osteoblasts or osteoclasts and are generally referred to as markers of bone turnover [10]. Bone resorption markers include tartrate-resistant acid phosphatase (TRAP) (enzyme produced by active osteoclasts), cathepsin K (protease responsible for collagen type I degradation), C-terminal (CTX) and N-terminal (NTx) telopeptides of type I collagen (fragments released during collagen breakdown). Bone formation markers include alkaline phosphatase (ALP/BALP) (enzyme linked to matrix mineralization), osteocalcin (OC) (non-collagenous protein secreted by osteoblasts), and procollagen type I N-terminal propeptide (PINP) (marker of new collagen synthesis). Additionally, cytokines and regulatory molecules such as RANKL, OPG, IL-1β, IL-6, and TNF-α modulate osteoclast and osteoblast activity, influencing the balance between bone formation and resorption [11].

N-telopeptide (NTx) is a terminal (telopeptide) fragment of type I collagen, released following the degradation of this collagen by osteoclasts. Type I collagen is the major component of bone matrix, and its degradation indicates bone resorption activity. This marker, evaluated in numerous basic research studies, increases significantly in the first 1–3 weeks after the application of orthodontic force, reflecting intense osteoclastic activity [11,12,13]. Knowledge of the level of N-telopeptide in GCF and its effects on cellular processes could provide applicable information regarding the evaluation of bone resorption phenomena that occur during orthodontic treatment.

Because orthodontic treatment typically extends over a period of 2–3 years and may lead to complications such as dental caries and periodontal deterioration, identifying non-invasive approaches to reduce treatment duration and improve therapeutic outcomes has become a major focus of research. One of the most effective strategies for shortening treatment time involves enhancing the rate of orthodontic tooth movement, a process largely dependent on osteoclast differentiation.

A series of studies have been conducted on the use of laser therapy during orthodontic movements; due to the laser biostimulation effect it generates during the remodeling process of soft and hard oral tissues [14,15,16]. Laser biostimulation of oral tissues induced by low-intensity lasers (LLLT) is related to the absorption of light by cells in the target tissues, resulting in an increase in cellular metabolism. At the cellular level, laser photons are absorbed primarily by cytochrome c oxidase in mitochondria, leading to increased production of ATP and nitric oxide (NO). This enhancement in cellular energy promotes osteoblastic and osteoclastic activity, accelerating bone remodeling [17]. Animal studies have shown that the use of laser therapy translates into better tooth movement and an increase in the rate of osteoclast formation in the area of tooth compression, reducing treatment time [18,19].

Although laser therapy is increasingly used in various medical fields, especially for its biostimulation effects and surgical applications, in orthodontics, there is still no standardized protocol for its use. Through this study, we aimed to evaluate the advantages and limitations of laser therapy in bone remodeling during orthodontic treatment by analyzing N-telopeptide levels in gingival crevicular fluid. The study hypothesis is that laser therapy might be used as a helpful adjuvant during the orthodontic treatment of adult patients, given that fact that it is more difficult compared to growing individuals.

## 2. Materials and Methods

### 2.1. Study Design and Ethical Considerations

This study was designed as a split-mouth observational research and received ethical approval from the Scientific Research Ethics Committee of George Emil Palade University of Medicine, Pharmacy, Science and Technology of Targu Mures, registration number: 1743/20 May 2022.

### 2.2. Patient Selection

The study included 30 adult patients with fixed orthodontic appliances selected from from CMI dr. Lazăr Luminița, in the following period: March–December 2023.

Inclusion criteria:

- Adult patients aged between 20 and 50 years;
- Orthodontic treatment with fixed appliances for dentoalveolar crowding;
- Maintaining a healthy periodontal status during orthodontic treatment.

The exclusion criteria were as follows:

- Poor oral hygiene during orthodontic treatment;
- Systemic disorders with potential impact on periodontal tissues (diabetes, immunological conditions, acute articular rheumatism, tuberculosis, etc.);
- Breastfeeding or pregnancy;
- Smoking;
- Use of antibiotics, vitamin D, or Ca supplements within the past 3 months;
- Previous use of anti-inflammatory drugs (NSAIDs).

The patients were informed about the study protocol, notified that they could withdraw at any time, and provided written informed consent.

### 2.3. Sample Size

For the primary comparison, a paired design was used (treated vs. control hemiarchs). α = 0.05, a sample of 30 pairs yields approximately 75% power to detect a medium effect size (Cohen’s d = 0.5).

### 2.4. Orthodontic Protocol

After the clinical examination, the patients diagnosed with dentoalveolar disharmony with crowding were subjected to fixed orthodontic treatment using brackets with an identical slot size (0.22) (American Orthodontics, Sheboygan, WI, USA). The sequence of arch wires used was NiTi size 0.12 at appliance placement, followed by 0.16 at the first activation, 0.16 × 0.16 at the second, and 0.16 × 0.22 at the third activation (BioForce, Dentsply Sirona, Charlotte, NC, USA).

To replicate standard clinical conditions, light continuous forces between 50 and 100 g were applied during each wire change. The magnitude of the force was measured and confirmed using a calibrated Correx tension gauge (Haag-Streit AG, Köniz, Switzerland) by documenting the deflection of the archwire immediately following ligation. This approach ensured consistent force application across all subjects and avoided extreme or outlier values.

### 2.5. Periodontal Protocol

During the orthodontic treatment, patients’ oral hygiene habits and periodontal status were monitored by recording the following indices: plaque index (PI), bleeding on probing index (BOP), and clinical attachment loss (CAL). Patients who presented inadequate hygiene at the activation sessions of the orthodontic appliance benefited from new training on oral hygiene measures. For patients who presented alterations in the periodontal status, we interrupted the active orthodontic treatment and instituted therapeutic measures corresponding to the degree of periodontal damage. The steps of the research methodology described below, as well as the laser protocol and the immunoenzymatic analysis, were performed only when a healthy periodontal status was confirmed.

### 2.6. Blinding

**Figure 1:**
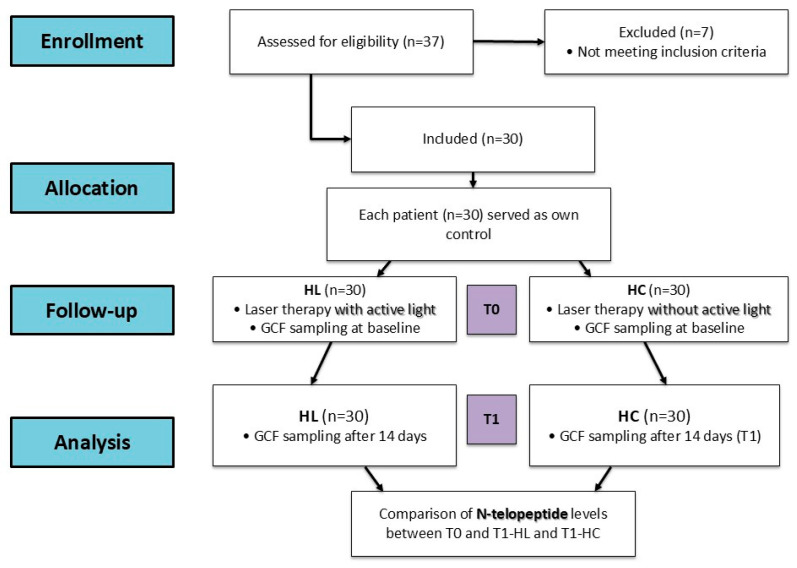
Flow diagram of the study following the CONSORT statement.

To minimize measurement bias, the examiner responsible for collecting and analyzing the gingival crevicular fluid samples was blinded to which hemiarch had been exposed to laser irradiation. All participants and investigators wore protective eyewear during laser use, and the device displayed identical visual indicators, preventing the examiner from identifying the treated side. The clinician applying the laser was not involved in either sample collection or data analysis, ensuring independent assessment of the outcomes (Figure 1).

### 2.7. Laser Protocol

Laser therapy was applied by a different member of the research team to a randomly selected hemiarch (HL) for each patient immediately after activation of the device (time T0, baseline). Laser therapy was applied to the tooth selected for GCF collection and to the neighboring teeth located mesially and distally. The same protocol was followed on the control hemiarch (HC) but without active light. Patients and operators wore protective goggles during exposure. Patients and team members who analyzed the GCF samples were not informed about the choice of HL and HC.

Laser therapy was performed with a dental laser (WISER3—Doctor Smile, Lambda S.p.A.), with a power of 12 watts, setting the Periodontology working mode with the following specifications: 1 W, 450 nm, 1 cm^2^ beam area, 15 s exposure time per point.

The laser was applied at the following sites: the distobuccal gingival crest, the mesiobuccal gingival crest, a central point on the buccal surface corresponding to these two sites, and two points at the tooth apex positioned parallel to points 1 and 2, respectively. The same sites on the palatal surface were selected for irradiation; consequently, the laser application was performed in 10 points per tooth, in contact with the gingival tissue. Each point was exposed for 15 s (energy = 15 J; fluence = 15 J/cm^2^). This resulted in a total of 150 J delivered per tooth per session, and for three teeth in each hemiarch, a total of 450 J per session. The protocol consisted of one every 14 days, yielding a cumulative dose of 150 J and 450 J per hemiarch. CW emission corresponds to a 100% duty cycle. Although the device is capable of delivering 12 W in surgical mode, in the Periodontology mode, the output parameters remain within the standards for LLLT.

### 2.8. Immunoenzymatic Analysis

Gingival crevicular fluid (GCF) samples were collected with special strips from the analysis kit, from the compression area secondary to orthodontic movement. The samples were taken prior to activating the orthodontic device (baseline, time T0) and after 14 days from the first activation (time T1) from both control and laser hemiarches (HL and HL). The GCF was not collected from the same tooth for all the patients. For each of them, the tooth that was the most affected by the orthodontic forces was chosen. The fluid was collected from the same tooth and baseline and at T1 (HL), using, of course, the same tooth from the opposing hemiarch (HC) as control. Thus, for each patient, we had three samples, one at baseline and two at T1 (HC, HL).

The patients gently rinsed their mouths with water, and then, before collecting the GCF samples, the teeth were isolated with cotton rolls, and the sampling area was dried with an air jet. The collection of GCF was conducted by using filter paper strips (Periopaper, ProFlow Inc., Amityville, NY, USA), which were placed into the gingival sulcus to a depth of about 1 mm and held for 30 s or until the moment their terminal part was fully saturated. The volume of crevicular fluid was assessed with the Periotron 8000 device (Pro-Flow Inc., Amityville, NY, USA), which measured the capacitance change of the filter paper. The strip was placed between the device’s two plates, and after the measurement, the result was read from the digital screen and registered in the datasheet. The acquired values were transformed into microliters utilizing a specialized software (mlconvert.exe—Ora Flow, http://oraflow.com/, Amityville, NY, USA). Following every measurement, the Periotron plates were sanitized using sterile compresses damped with saline in order to avoid contamination. The paper strips displaying evidence of blood were eliminated.

Subsequent to reading the results of each paper strip, these were placed into Eppendorf safe lock cryotubes that were filled with 250 μL of phosphate-buffered solution. The samples were then placed in compartmented cryoboxes and maintained at a temperature of −80 °C until immunoenzymatic processing. The GCF specimens were tested without dilution, in accordance with the protocol.

N-telopeptide level determination (AssayGenie, Dublin, Ireland), an indicator of the bone resorption process, was conducted using the enzyme-linked immunosorbent assay (ELISA) procedure, under the immunology laboratory conditions of CAMPhR, of G. E. Palade UMPHST of Targu Mures.

The reagents were allowed to reach room temperature and prepared following the protocol. After the thawing of the GCF specimens, they were vortex-mixed (Vortex ZX4, Velp Scientifica, Usmate, Italy) for 60 s, and then centrifuged (Eppendorf MiniSpin Centrifuge, Hamburg, Germany) at 10,000 rpm for 2 min.

During the initial phase, the standards and samples were introduced into the wells, which were coated with an antibody targeting N-telopeptide specifically. Throughout the incubation cycle, the N-telopeptide contained in the standard or the sample attached to the antibody, which was immobilized, and created an antibody-antigen complex. Following the incubation step, the N-telopeptide from the standard or the sample that was not bound to the fixed antibody was eliminated through aspiration, rather than washing.

The next step from the protocol involved incorporating a detection antibody that specifically targets the N-telopeptide and which is labeled with biotin. This was followed by a period of incubation, allowing the detection antibody to attach to the N-telopeptide found in the standard and sample, which was captured by the antibody immobilized in the well, creating the antibody–antigen–antibody complex (the “sandwich” principle). Following incubation, the specific detection antibody that was not bound was eliminated by rinsing with a washing buffer solution.

The third step of the method consisted of adding the conjugate (avidin–HRP: avidin is a protein labeled with an enzyme—horseradish root peroxidase), and during the incubation, it bound to the specific detection antibody labeled with biotin. After the incubation period, the washing process was followed again with the removal of the unbound avidin.

Subsequently, the substrate was added (a solution that contains a chromogenic compound—tetramethylbenzidine), followed by an incubation period, which reacted with the conjugate, producing a color reaction (blue), depending on the amount of N-telopeptide bound in the standard or sample. After the stopping solution containing sulfuric acid was added, the reaction color shifted from blue to yellow.

The absorbance of each well was measured spectrophotometrically using the Dynex DSX automated ELISA analyzer (DYNEX Technologies, Chantilly, VA, USA). The analyzer software generated a standard curve by comparing absorbance values with the known concentrations of the standards. The N-telopeptide concentration in each sample was then determined by comparing its absorbance to this calibration curve, with the concentration being directly proportional to the color intensity of the well. According to the volume of phosphate-buffered saline (PBS) used for dilution and the gingival crevicular fluid volume recorded with the Periotron, the measured N-telopeptide concentrations were adjusted.

Performance characteristics:

- Kit sensitivity: detection limit for telopeptide is 0.15 ng/mL;
- Measurement range: 0.312–20 ng/mL (all samples fell within this range);
- Kit precision: the precision intra-assay has a coefficient of variation below 5.5%; inter-assay precision has a coefficient of variation below 7.9%.

#### Laser Therapy Evaluation

To assess the effects of laser therapy, the values obtained for N-telopeptide were compared between HC and HL.

### 2.9. Statistical Analysis

Data were compiled using Microsoft Excel (Microsoft Corporation, 2018, Redmond, WA, USA). Statistical analyses were performed with GraphPad Prism version 8.0.0 for Windows (GraphPad Software, San Diego, CA, USA). Descriptive statistics, including the mean, standard deviation, median, minimum, and maximum values, were calculated for each dataset. The normality of data distribution was assessed using the Kolmogorov–Smirnov test. Differences in N-telopeptide values between baseline (T0) and T1 were evaluated using the Wilcoxon signed-rank test for non-normally distributed data and the paired *t*-test for normally distributed data. Comparisons between HC and HL at T1 were performed using the paired *t*-test. Effect sizes were calculated as follows: for Wilcoxon signed-rank tests, the effect size r was computed as r = Z/√N, where Z is the standardized test statistic; for paired *t*-tests, effect size was expressed as Cohen’s d for dependent samples, computed as: d = (M1 − M2)/SD_diff, where SD_diff is the standard deviation of the difference scores. Interpretation of effect sizes followed Cohen’s guidelines: Wilcoxon effect size r: small (r ≈ 0.10), medium (r ≈ 0.30), and large (r ≥ 0.50), Cohen’s d (paired samples): small (r ≈ 0.20), medium (r ≈ 0.50), and large (r ≥ 0.80). The correlation between paired measurements (baseline vs. T1) was calculated using Pearson’s correlation coefficient to assess the strength of pairing prior to *t*-test analysis. A *p*-value < 0.05 was considered statistically significant.

## 3. Results

The following formula was used to determine N-telopeptide values in gingival crevicular fluid:

Total protein concentration in original crevicular fluid (ng·mL^−1^) was calculated as follows:

$$C_{\mathrm{original}}=\frac{C_{\mathrm{diluted}}\times V_{\mathrm{diluted}}}{V_{\mathrm{sample}}}$$

where $C_{\mathrm{diluted}}$ is the protein concentration measured in the diluted sample (ng·mL^−1^), $V_{\mathrm{diluted}}$ is the total volume of the diluted sample (mL; e.g., 0.25 mL), and $V_{\mathrm{sample}}$ is the volume of the crevicular fluid originally collected (mL).

Mean values of N-telopeptide (ng/mL) at time T0 and T1 (HC, HL) are included in Table 1.

**Figure 2:**
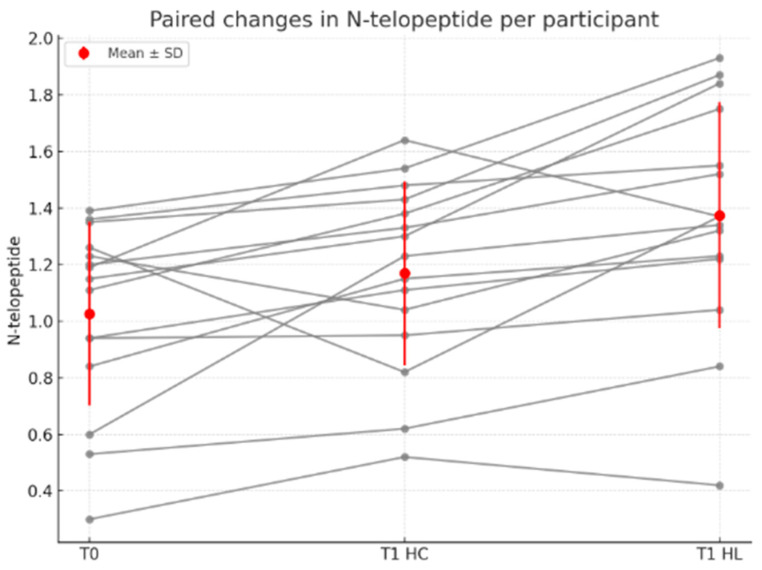
Distribution of N-telopeptide level (ng/mL) at T0 and T1 for HC and HL.

**Figure 3:**
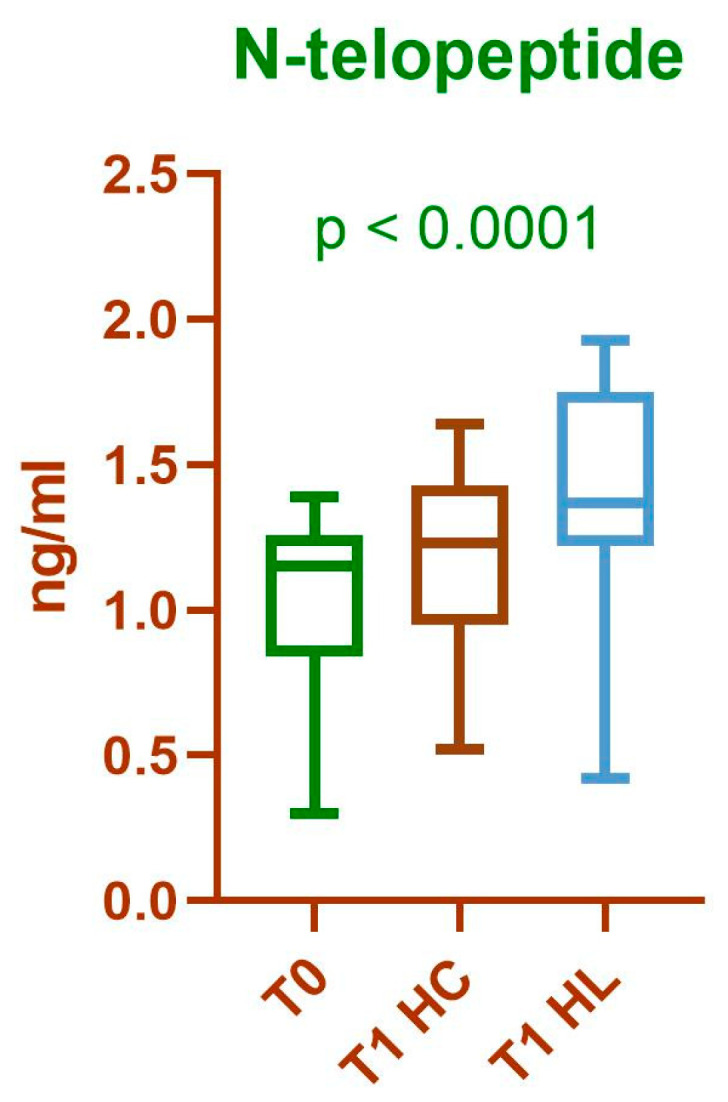
Comparison of N-telopeptide values at time points T0 and T1 for HC and HL. Results are expressed as median and interquartile range; *p*-value was calculated using the Friedman test.

Comparing the N-telopeptide values at T0 and T1 for HL, using the Wilcoxon test, we obtained a statistically significant difference (*p* < 0.0001, median difference = 0.3100, 95% CI 0.240–0.370, N = 30). The effect size was large (r ≈ 0.91). A statistically significant difference was detected when comparing the N-telopeptide values in HC between baseline and T1 using the Wilcoxon test (*p* = 0.0018, median difference = 0.1500, 95% CI 0.060–0.250, *p* = 0.0018, N = 30). The effect size was moderate to large (r ≈ 0.57). A statistically significant difference was observed between HC and HL at T1, using the paired *t*-test (*p* < 0.0001, mean difference = 0.2047, SD = 0.2256, 95% CI 0.1204–0.2889, t (29) = 4.970, *p* < 0.0001, N = 30). The effect size was large, as indicated by Cohen’s d for dependent samples (d = 0.91) and the correlation between paired measurements was strong (r = 0.8261), indicating effective pairing (Figure 2 and Figure 3). Overall, the large effect sizes observed across tests suggest that laser application produced a biologically relevant increase in N-telopeptide levels, reflecting a substantial stimulation of bone resorption activity in the treated hemiarch.

## 4. Discussion

To assess the effects of laser therapy during orthodontic treatment in adult patients, we compared the levels of N-telopeptide in the hemiarches treated with laser (HL) and in the control (HC) hemiarches.

In a previous study, we evaluated laser therapy during orthodontic treatment by recording clinical indicators [20]. It is proven that the determination of markers in GCF can be a more objective monitoring method than a clinical one [21,22,23]. Therefore, in the present study, we tried to validate the use of laser therapy as an adjuvant treatment during orthodontic treatment by determining N-telopeptide in GCF.

N-telopeptide is a specific marker of type I collagen degradation and osteoclastic activity and was identified in the gingival crevicular fluid in progressive increase during the period 7–21 days after orthodontic activation [12,24].

Based on these observations, we chose to collect N-telopeptide samples from the pressure zone, where bone resorption phenomena predominate, 14 days after activation of the orthodontic appliance, at which time their levels would be higher in the GCF.

The N-telopeptide values recorded at time T1 were higher than those recorded at baseline, both in HC and HL. The differences were statistically significant, *p* < 0.0001, in HL and *p* = 0.0024, in HC. However, only for 2 of the 30 patients, these values were higher for samples collected from HC than those collected from HL. N-telopeptide, a marker of bone resorption, showed a tendency to increase in the gingival crevicular fluid during orthodontic treatment, which suggests an increased activity of osteoclasts during orthodontic movement, favoring bone resorption. The fact that N-telopeptide levels were higher in HL compared to HC demonstrates the effectiveness of laser therapy as an adjuvant in bone remodeling.

Similar results to our study were obtained by Alnazeh et al. The authors evaluated cytokine levels and bone metabolic biomarkers in patients receiving fixed orthodontic therapy and Invisalign. Regarding bone metabolic biomarkers, it was shown that N-telopeptide levels increased significantly (*p* < 0.05) without differences between the investigated groups [21].

Other authors obtained different results from those mentioned above. Kloukos et al. investigated the C-terminal telopeptide of type I collagen (CTX) and the N-terminal pro-peptide of type I pro-collagen (PINP) in GCF and blood serum of patients with fixed orthodontic appliances, comparing the changes in these markers during early stages of orthodontic treatment. No statistically significant changes in CTX or PINP levels were observed in either GCF or serum during the first 14 days of orthodontic treatment. This suggests that initial appliance placement may not cause significant local or systemic changes in bone turnover during this early phase [25].

A series of studies have evaluated the effects of laser therapy on orthodontic movements and levels of some proinflammatory cytokines in GCF. Prathapan Santhakumari et al. observed that intermittent laser therapy for 12 weeks led to significant increases in cytokine levels (IL-6, TNF-α, and IL-1β) in the study group compared to the contralateral control group [26]. A similar study investigated the effects of low-level laser therapy on interleukin-1β (IL-1β) levels in gingival crevicular fluid and its correlation with orthodontic tooth movement. The findings indicated that low-level laser therapy elevated IL-1β levels in the gingival crevicular fluid and enhanced the rate of orthodontic tooth movement [27].

The determination of these markers in GCF is an objective method to assess the effects of laser therapy as an adjuvant in orthodontic treatment. However, it is important to recognize that the values of these proinflammatory markers may also be affected by other factors such as inadequate oral hygiene or gingival inflammation. Numerous studies have shown that their levels in GCF are elevated in sites with periodontal conditions compared to healthy ones [28,29,30,31].

Therefore, we believe that the use of bone turnover markers, such as N-telopeptide, can provide more precise information on the efficiency of laser therapy on bone remodeling phenomena during orthodontic treatment.

Due to the fact that orthodontic treatment in adult patients differs markedly from that in children and adolescents, it would be useful to rely on alternative approaches that emphasize dentoalveolar compensation. Laser therapy can be used as an adjuvant during orthodontic treatment in this sense [1]. A study by Huang et al. has shown that LLLT markedly enhances osteoclast formation, which is essential for bone resorption. LLLT has been shown to stimulate osteoclast differentiation in a manner dependent on RANKL and to increase the expression of osteoclast-related functional genes—such as CTSK, TRAP, NFATc1, or MMP9—in RAW264.7 cells. In addition, the involvement of the NF-κB signaling pathway in LLLT-induced osteoclastogenesis has been verified. Clinically, LLLT has been reported to enhance cumulative tooth movement and modulate bone density loss, highlighting its potential as an effective adjunct in orthodontic therapy [32].

In a study by Gonçalves et al., LLLT has been shown to exert a biostimulatory effect on bone remodeling by enhancing the proliferation and differentiation of osteoclastic, osteoblastic, and fibroblastic cells. This therapeutic approach has demonstrated its ability not only to accelerate orthodontic tooth movement (OTM) but also to reduce external root resorption, regulate the inflammatory response, and alleviate pain and discomfort associated with OTM [33].

The limitations of this study arise from the small sample size, the collection of GCF samples at different points during the orthodontic treatment, and the relatively broad age range of participants. Although the wide age range (20–50 years) may be viewed as a drawback, we consider it beneficial for enhancing the generalizability of our findings to future treatment guidelines. Nevertheless, combining a broad age distribution with varying treatment stages at the time of laser application introduces another constraint. Both factors can influence bone metabolism and tissue responsiveness to laser biostimulation, which may, in turn, affect the levels of the biochemical markers analyzed. The fact that only one sample on GCF was collected at baseline could also constitute a limitation. This was done considering that there were no major differences between the two sides at that time, and in order to be able to include more patients in the study. The measurement of only one biochemical marker, N-telopeptide, reflecting bone resorption, without including markers of bone formation (for example, osteocalcin, PINP) can also be considered a limitation.

## 5. Conclusions

Measuring N-telopeptide levels in gingival crevicular fluid offers an objective way to assess the remodeling of periodontal tissues during orthodontic treatment.

In this study, the laser-treated hemiarches showed higher N-telopeptide levels than the controls, indicating that laser biostimulation may temporarily increase bone resorption activity. However, because the study evaluated a single biochemical marker at one time point (14 days) and did not measure pain levels, post-treatment stability, and tooth movement rate, additional research is required to determine its clinical relevance and to establish standardized protocols for incorporating laser biostimulation into orthodontic therapy.

## Figures and Tables

**Table 1:** N-telopeptide levels measured at baseline and T1 for HC and HL.

Mean Value and Standard Deviation for Identified Protein (ng/mL) T0	Mean Value and Standard Deviation for Identified Protein (ng/mL) T1—HC	Mean Value and Standard Deviation for Identified Protein (ng/mL) T1—HL
0.1026 ± 0.3247	1.169 ± 0.3247	1.374 ± 0.4001

## Data Availability

The original contributions presented in this study are included in the article. Further inquiries can be directed to the corresponding author.
